# Reducing the Risk of Postoperative Problems With Panniculectomies Using the Prevena Plus™ 125 Incisional Management Dressing

**DOI:** 10.7759/cureus.9341

**Published:** 2020-07-22

**Authors:** Michael N Desvigne

**Affiliations:** 1 Plastic Surgery, Abrazo Arrowhead Hospital, Glendale, USA

**Keywords:** prevena, panniculectomy, ventral hernia, obesity, preventing complications, negative pressure therapy, abdominal surgery, abdominal reconstruction, closed incisional negative pressure wound therapy, surgical incisions

## Abstract

Abdominal wall reconstruction procedures have become increasingly popular in recent years as technology and surgical techniques have improved. The downside to these procedures has been the high rate of postoperative complications. Surgical site infections have been reported as high as 33.7% of the $9.8 billion spent annually on these complications.

I present the case of a 62-year-old morbidly obese woman who underwent a combined procedure of abdominal wall reconstruction and panniculectomy. A total of 45 lbs of pannus was removed through a transverse incision that extended from hip to hip, measuring 90 cm in length. Following panniculectomy, abdominal wall reconstruction was performed by mobilizing the abdominal skin flap from the lower abdominal panniculectomy incision (avoiding a T-shaped incision with a traditionally high risk of dehiscence), and placement of biologic mesh as an underlay followed by fascial closure. Prevena Plus™ 125 (3M + KCI, San Antonio, TX) was applied for postoperative closed incisional negative pressure therapy (ciNPT) and continued for 10 days. No postoperative complications occurred. The incision healed without incident with no hernia recurrence at one year.

ciNPT in high-risk patients can help minimize the risk of postoperative wound healing complications and should be considered in high-risk patients. Those patients undergoing combined procedures and especially morbidly obese patients undergoing combined abdominal wall reconstruction and panniculectomy are at particularly high risk for wound healing complications. ciNPT should be considered as a postoperative dressing of choice in this challenging patient population.

## Introduction

Surgical complications, such as infection, seroma, and wound healing delay, are costly for health systems, challenging for surgeons, and worrisome for patients. Preventing problems is always preferred when possible. Abdominal wall reconstructive procedures with or without panniculectomy have become increasingly popular in recent years but the high rate of complications has encouraged surgeons to improve surgical techniques and consider postoperative dressings to assist with reducing these complications. Incisional management with negative pressure therapy has become a postoperative therapy utilized in multiple surgical procedures that carry a high risk of complications. The use of Prevena™ (3M + KCI, San Antonio, TX) for incisional management for abdominal wall reconstruction has been utilized and described [1,2]. However, less has been written describing the benefits of Prevena™ in the combination of abdominal wall reconstruction and panniculectomy, which carries a particularly high risk of postoperative complications. While Prevena™ is known to assist with reduction of lateral tension, edema control, and reduction of bacterial burden, the improved skin flap perfusion may be of particular benefit in these patients [2]. The use of Prevena™ and Prevena Plus™ 125 customizable for high-risk patients has been widely accepted and perhaps should become the postoperative dressing of choice in morbidly obese patients undergoing the combination of abdominal wall reconstruction and panniculectomy.

## Case presentation

**Figure 1:**
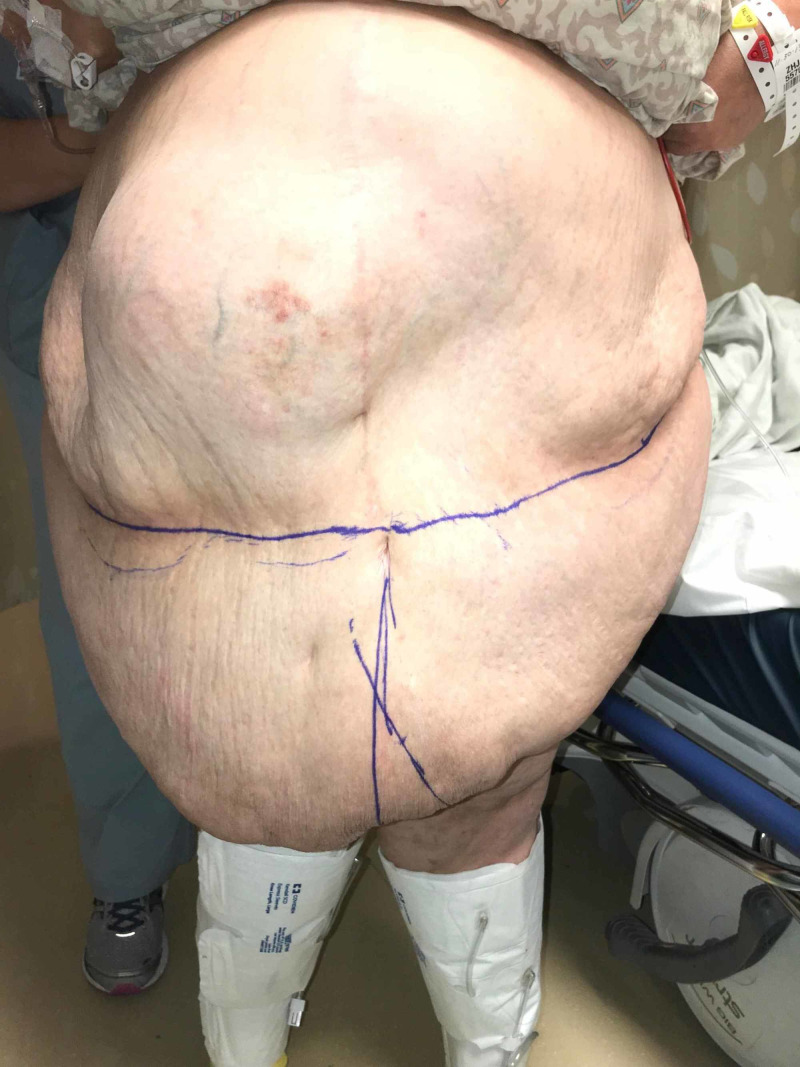
Preoperative photo

I present the case of a 62-year-old female nurse with morbid obesity (BMI 52) and a large ventral hernia (Figure 1).

She had undergone previous gastric bypass surgery and lost over 100 lbs and still weighed 320 lbs with a current BMI of 52. Despite her obesity, she was non-diabetic with no known cardiac disease. She presented to me as a referral from general surgery as she had a large recurrent ventral hernia as well as an excessive pannus. She had undergone previous open ventral hernia repairs with synthetic mesh twice. The pannus was not addressed during the hernia repairs or after her gastric bypass procedure. The weight of the pannus may have contributed to the hernia recurrence after repair, and ultimately compromised her functional status rendering her essentially unable to exercise and barely able to ambulate.

Preoperatively, the patient was optimized by her primary care physician. Following a lengthy discussion with the patient as well as her general surgeon, we all agreed that the best option was to proceed with one surgery to include both abdominal wall reconstruction with biologic mesh ventral hernia repair and panniculectomy to allow one anesthesia and one recovery. While we anticipated postoperative incisional wound healing complications and prepared the patient to expect such, we planned to utilize negative pressure incisional management with Prevena™ to reduce the risk.

**Figure 2:**
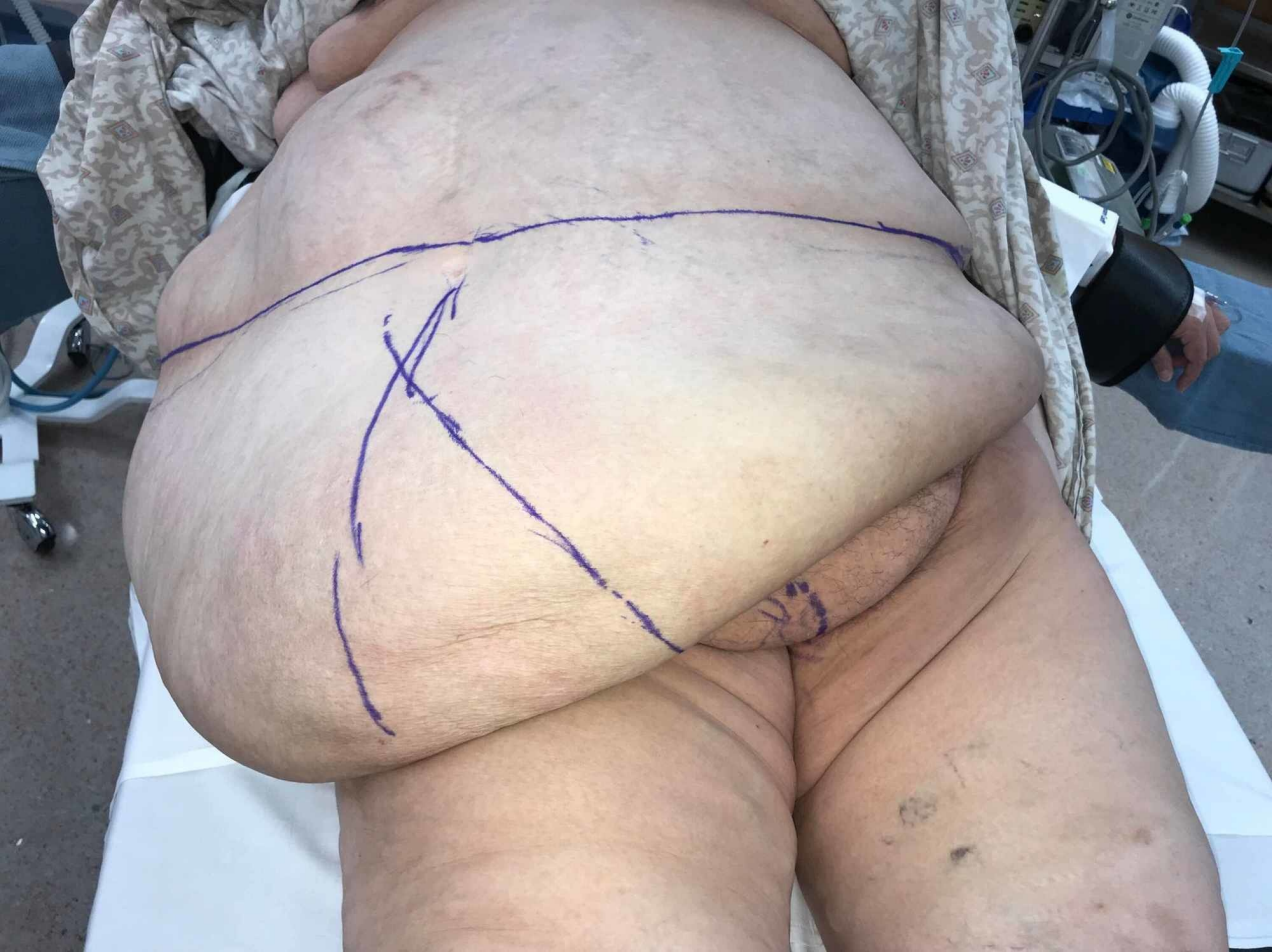
Large pannus with markings at midline and area for planned excision

Operatively, she underwent combined procedures including abdominal wall reconstruction with biologic mesh hernia repair and panniculectomy (Figure 2).

**Figure 3:**
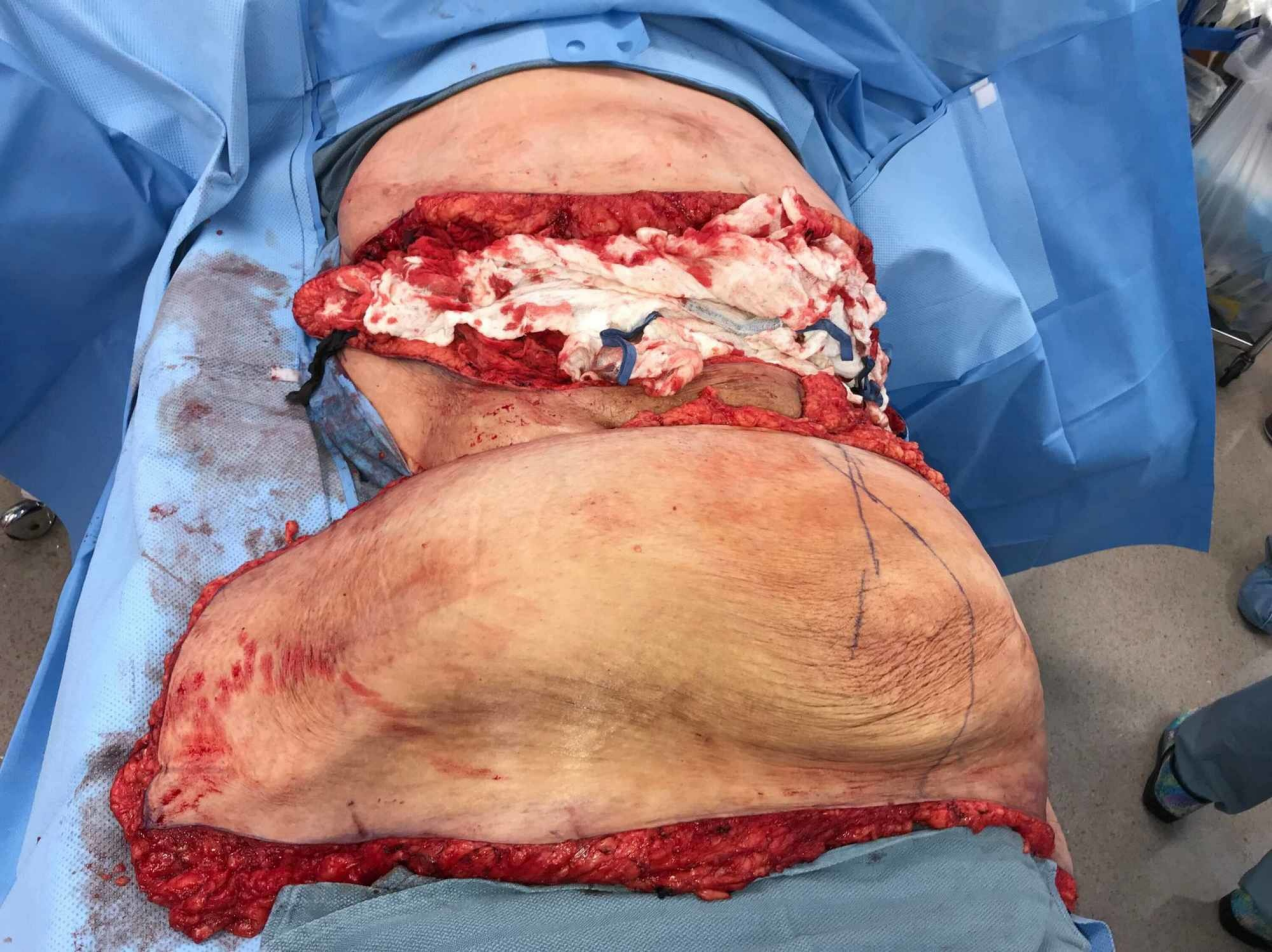
Pannus excised

**Figure 4:**
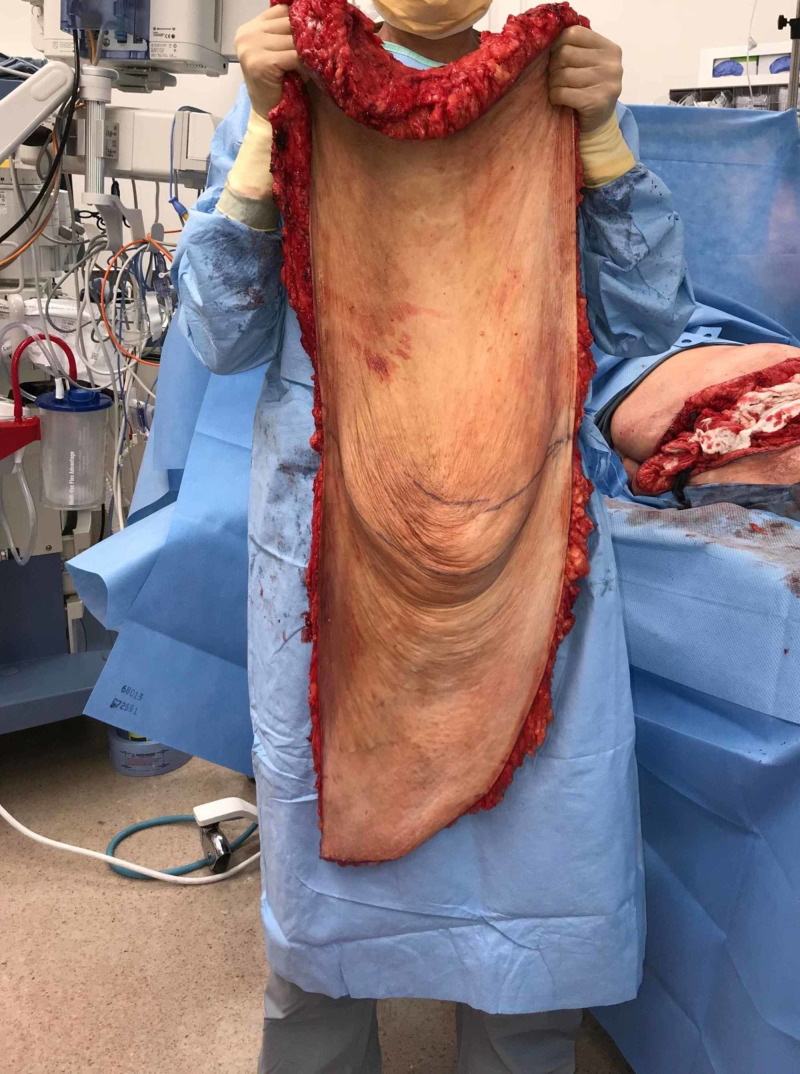
Pannus weighing 45 lbs

The large pannus was addressed first. An incision was made hip to hip measuring 90 cm. The excision included approximately 45 lbs of tissue (Figures 3, 4).

**Figure 5:**
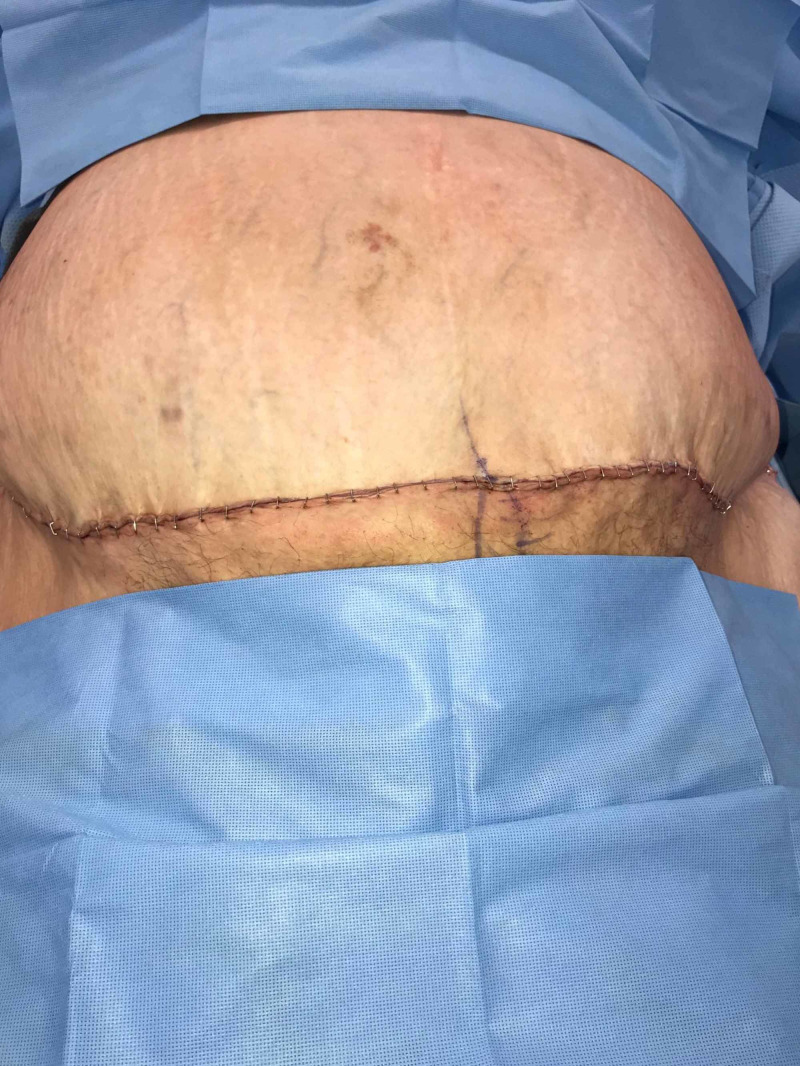
Incision closed with skin flaps appearing well perfused

The large hernia was then addressed and approached from the lower abdominal tranverse incision. An additional midline incision was avoided. The skin flap was raised, similar to an abdominoplasty flap. This allowed entrance to the hernia defect from a "new" tissue plane that enhanced our ability to address the hernia. Additionally, this avoided the midline T-shaped incision that historically is known to result in high rates of dehiscence. The hernia defect was identified, and biologic mesh was placed as an underlay and secured with a #0 polydioxanone monofilament absorbable suture. A layered closure was performed to include epidermal closure with skin staples. There was little to no tension along the incision, and the skin flaps appeared well perfused (Figure 5).

**Figure 6:**
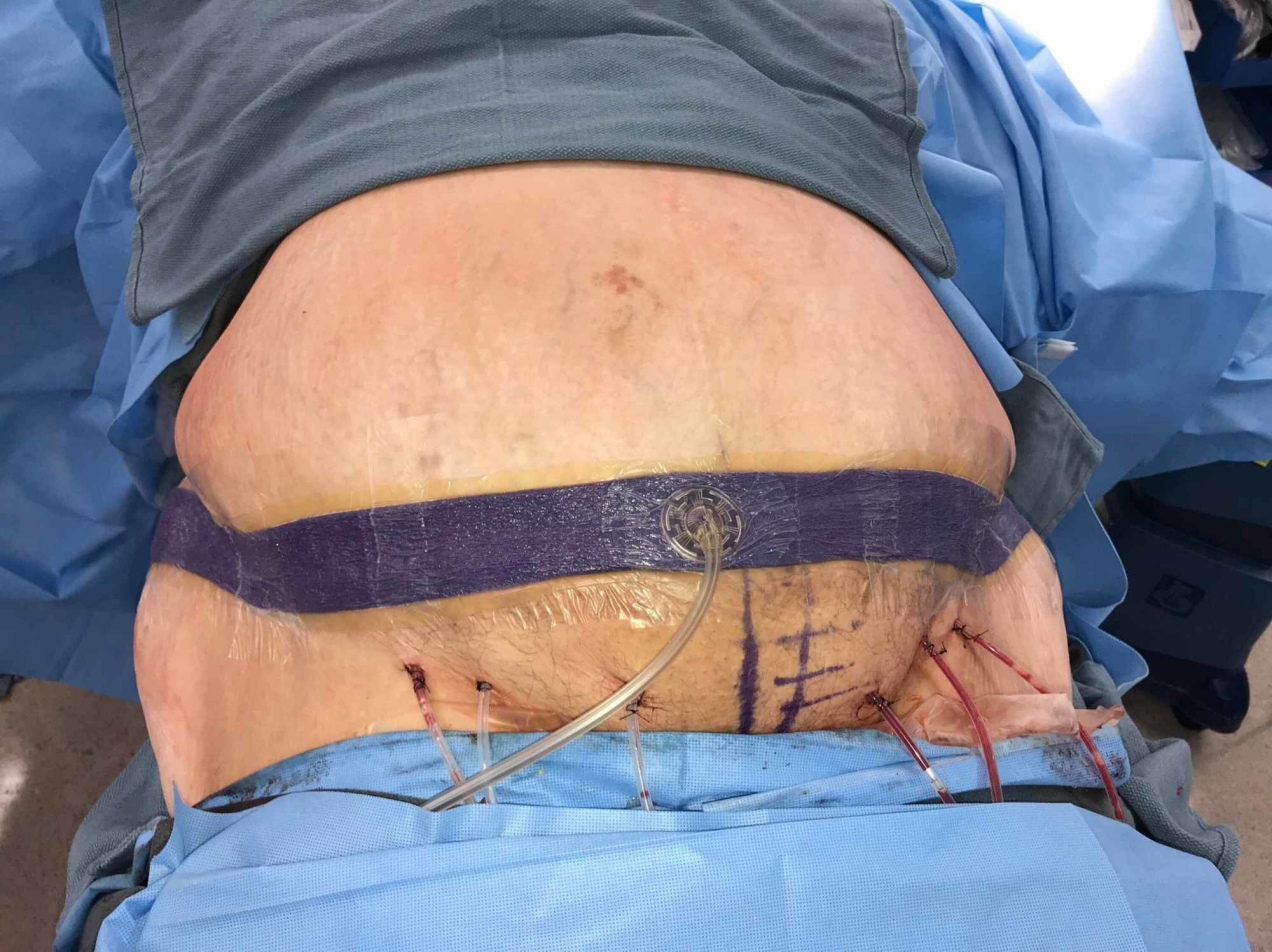
Prevena Plus™ 125 placement

Immediately following closure, Prevena Plus™ 125 was placed (Figure 6). The customizable dressing allowed for complete coverage of the incision (90 cm length).

**Figure 7:**
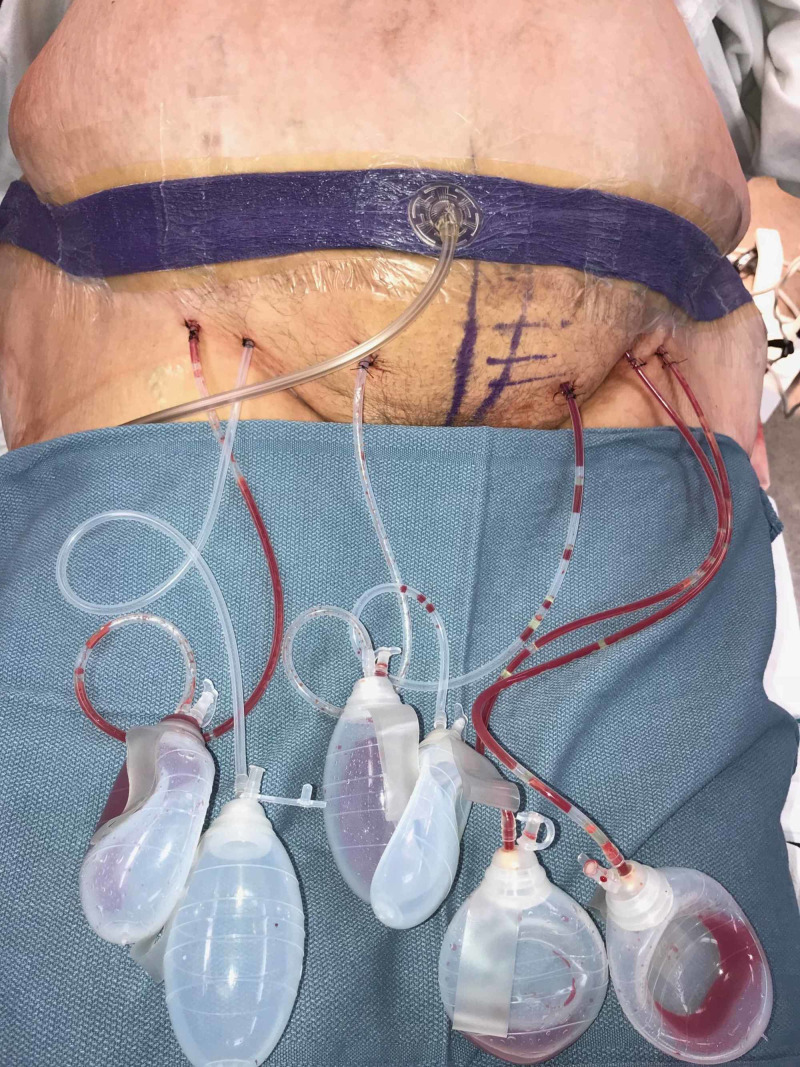
Six Jackson-Pratt drains placed

Because of the excess tissue removal (pannus) as well as the extensive undermining, six 10-mm Jackson-Pratt (JP) drains were placed (Figure 7).

The Ulta™ hospital VAC® unit (3M + KCI) was connected to the Prevena™ and utilized as the pump while the patient was hospitalized.

**Figure 8:**
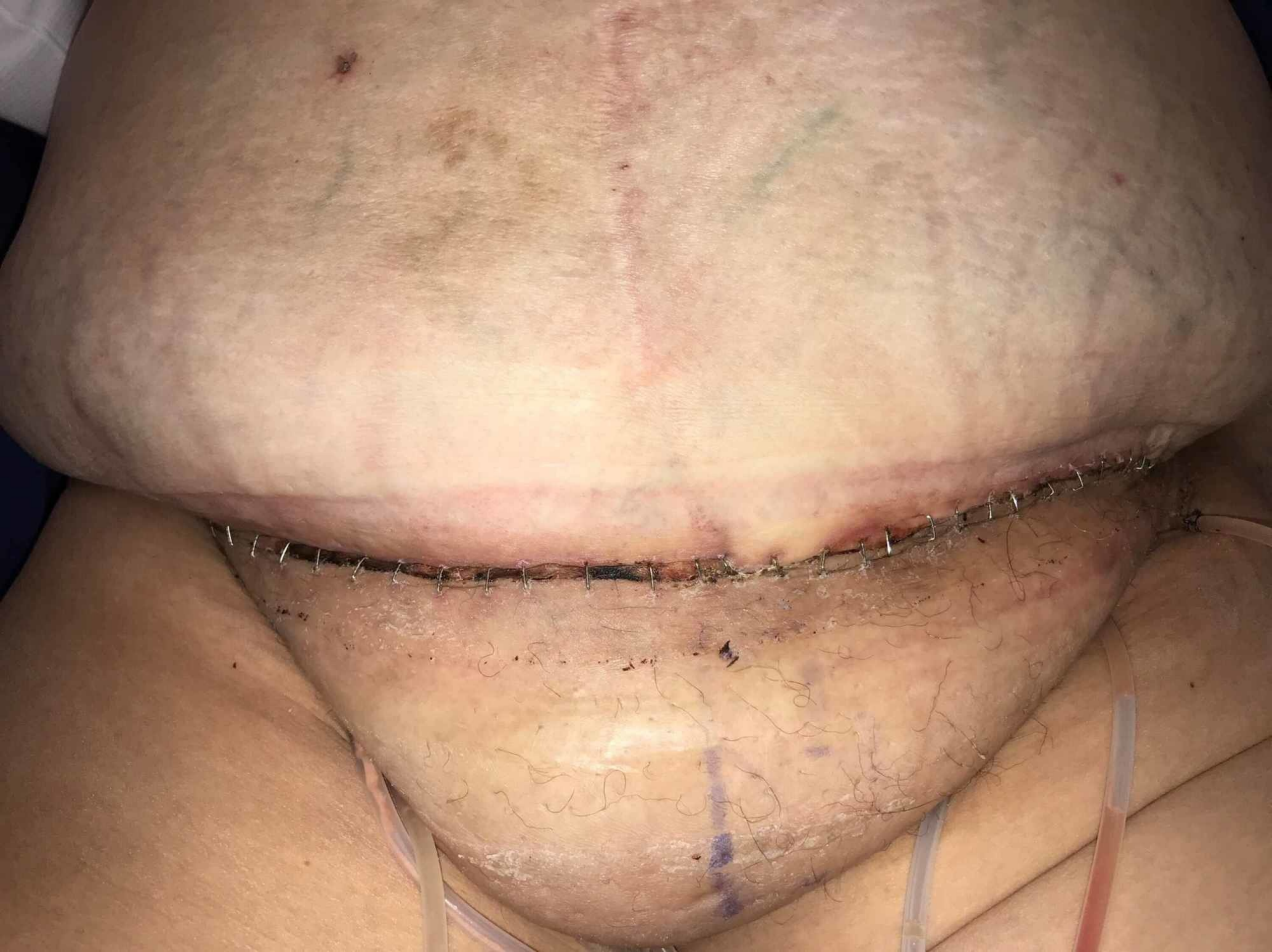
Anterior incision, Day 10

**Figure 9:**
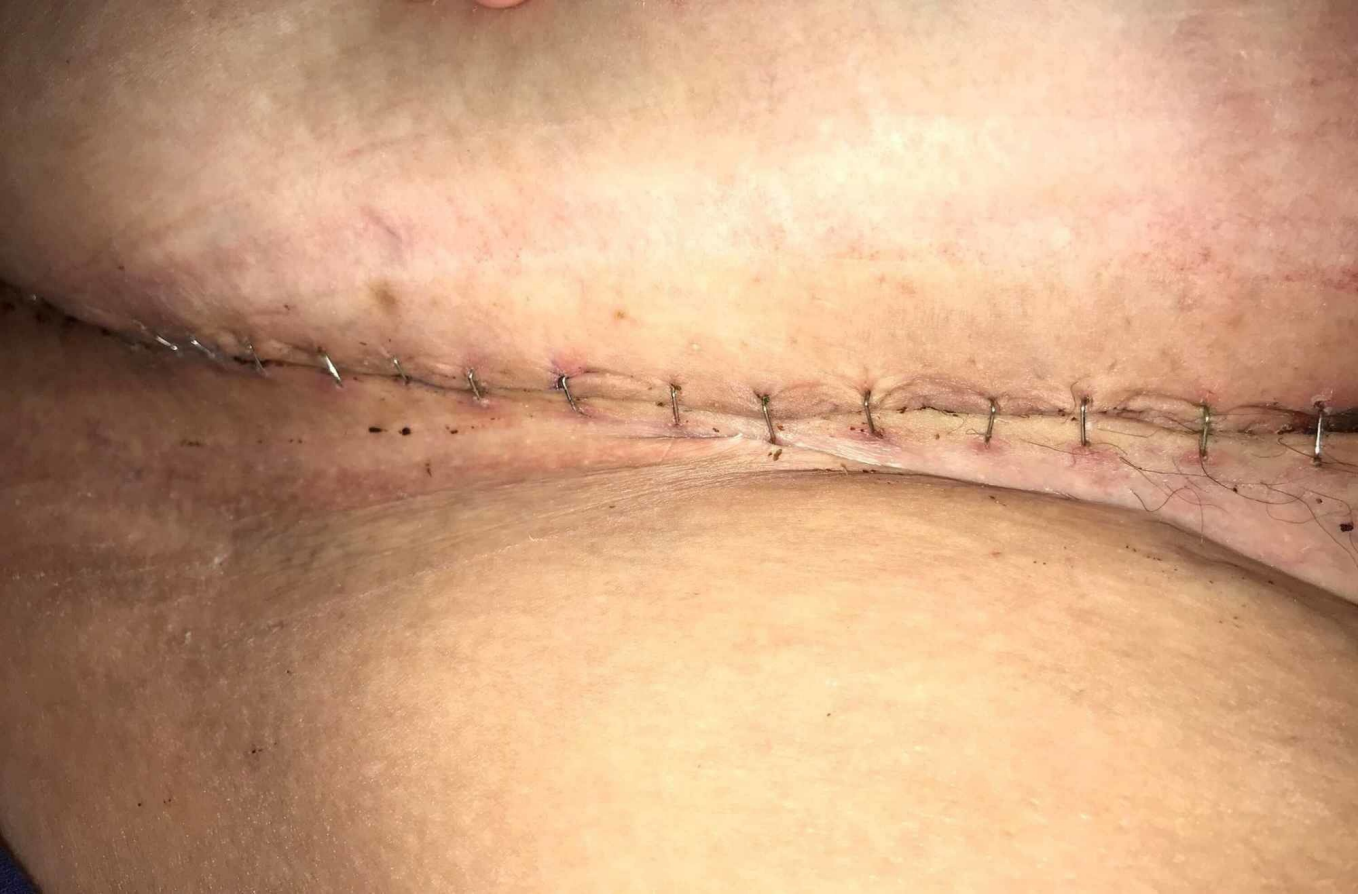
Incision on the right side, Day 10

**Figure 10:**
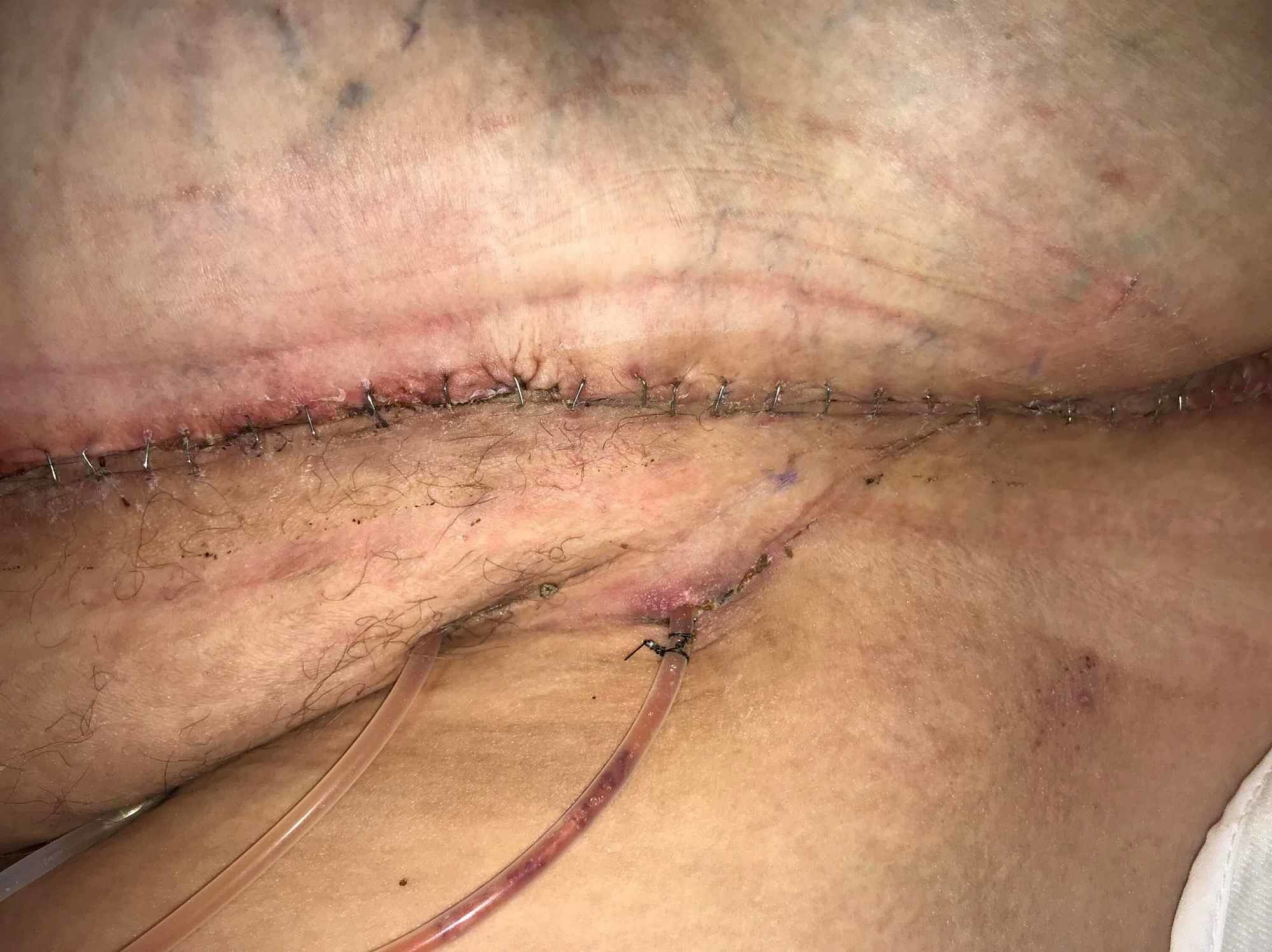
Incision on the left side, Day 10

Postoperatively, the patient remained in the hospital for 72 hr. Once she was ambulating, an abdominal binder was placed, the portable Prevena™ unit replaced the Ulta system, and she was discharged to home. On Day 10, the Prevena™ dressing was removed. The incision was intact. The dermal edges were well approximated and there was no drainage from the incision or evidence of infection (Figures 8-10).

**Figure 11:**
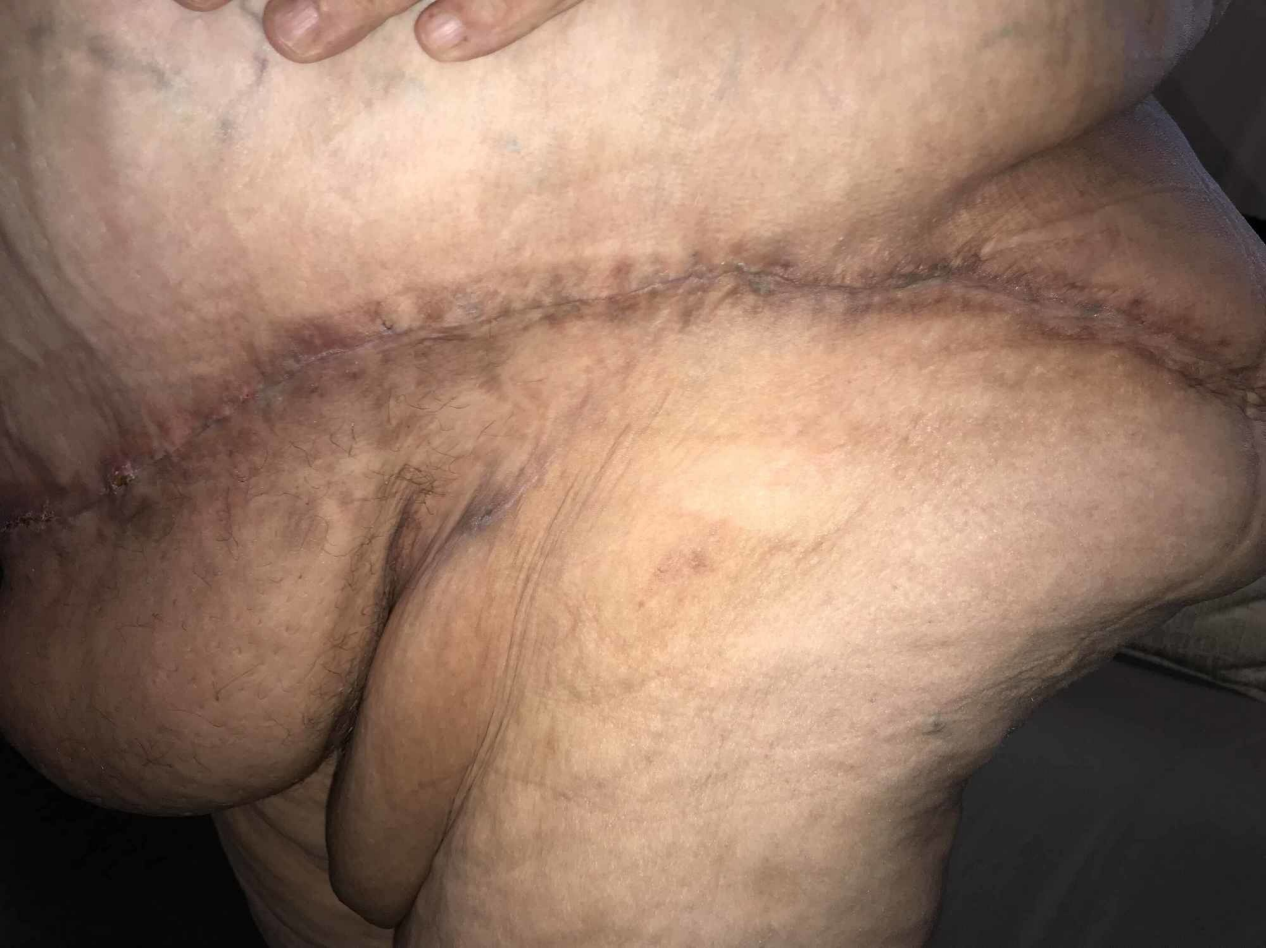
Incision on the left side, at six weeks

**Figure 12:**
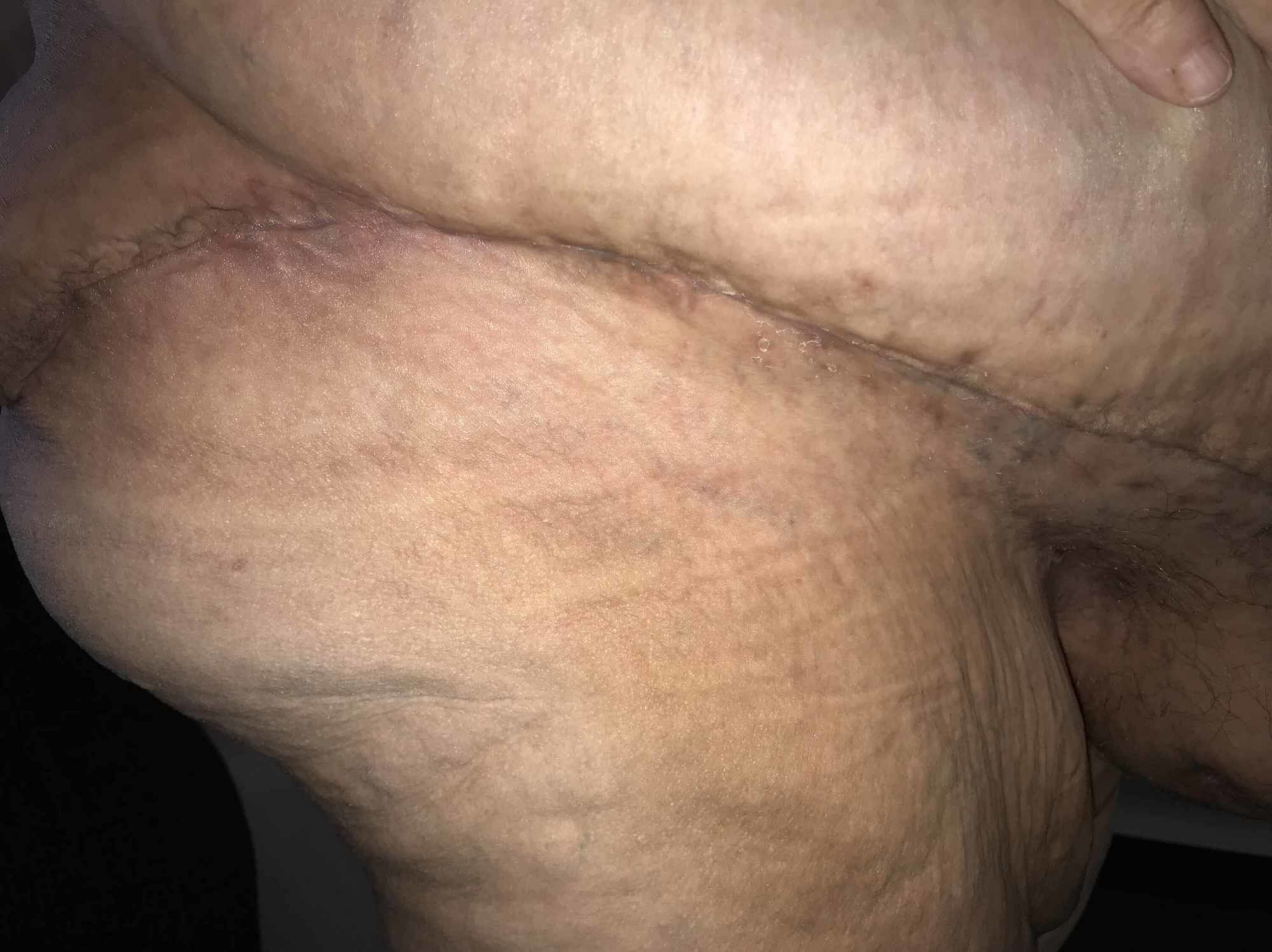
Incision on the right side, at six weeks

The JP drains were draining serosanguinous fluid and were left in place and removed when each JP output was less than 24 cc in 24 hr (1 cc/hr). At the end of six weeks, all the JP drains had been removed. The incision healed without incident (Figures 11, 12).

**Figure 13:**
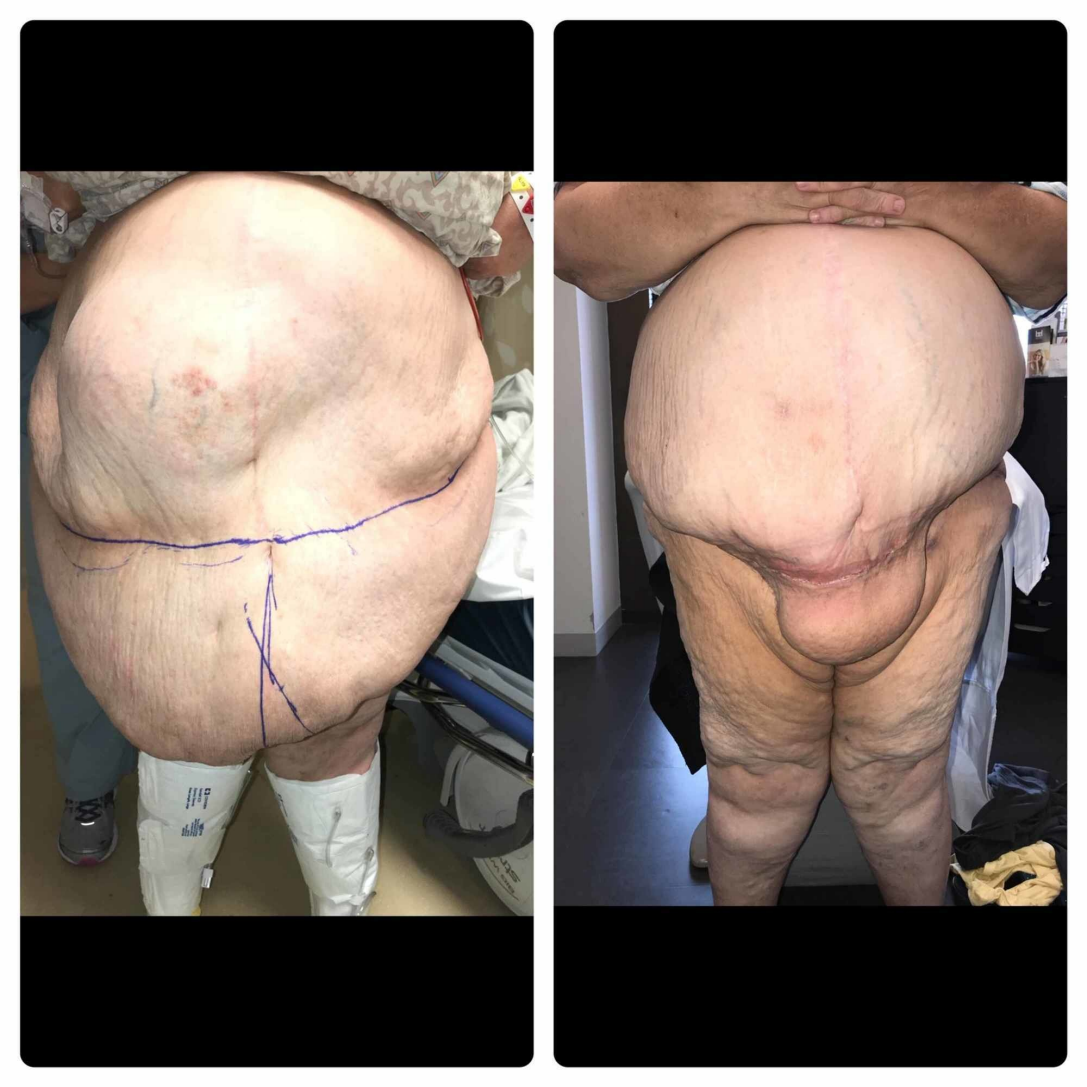
Before/After pictures, at six weeks

This was life changing for her, as she was finally able to walk and exercise (Figure 13).

The patient was followed up for one year with no evidence of incisional breakdown, seroma, or hernia recurrence. One year after her procedure, she had lost an additional 75 lbs, and resumed daily exercise.

## Discussion

Negative pressure wound therapy has become a new standard treatment for complex wounds since first introduced by Morykwas and Argenta in 1997 [3,4]. The physiologic response of the tissues to include macrostrain and microstrain has been well studied and described in the literature to account for a mechanism of action to assist in wound bed preparation, granulation tissue formation, and reduction in wound size and depth [4,5]. The use of negative pressure over a closed incision was described as early as 1997 in a porcine model. Stannard et al. published two prospective articles in 2006 and 2009 showing the benefit of negative pressure incisional management in acute orthopedic trauma [6,7]. Since then, a growing body of surgical literature has evolved that supports the use of negative pressure in closed incisions to decrease the incidence of surgical site infections and dehiscence. Multiple case series consistently noted a reduction in postoperative infection and surgical dehiscence when Prevena™ incisional management was utilized [6-8].

Closed incisional negative pressure therapy (ciNPT) has become more popular in recent years and used selectively in those cases where the risk of postoperative incisional complications is high. Singh et al. did a meta-analysis showing the clinical benefit of Prevena™, specifically comparing it to other devices on the market [9]. In 2019, Prevena™ received FDA approval to be used to reduce the risk of postoperative infections and dehiscence. The FDA specifically states,

PREVENA 125 and PREVENA PLUS 125 Therapy Units manage the environment of closed surgical incisions and remove fluid away from the surgical incision via the application of -125mmHg continuous negative pressure. When used with legally marketed compatible dressings, PREVENA 125 and PREVENA PLUS 125 Therapy Units are intended to aid in reducing the incidence of seroma and, in patients at high risk for post-operative infections, aid in reducing the incidence of superficial surgical site infection in Class I and Class II wounds. [10]

As Prevena™ has been utilized in a variety of surgical cases, the indications support the selective use of Prevena™, specifically, in those cases which have a high risk of postoperative complications such as infection, dehiscence, and/or seroma. Some of these patients and/or surgeries can be identified as high risk upon preoperative evaluation. Some are recognized as such while in the operating room where the proposed technique may be particularly challenging and/or the patient’s tissues are such that the anticipation of an incisional complication is high.

The importance of selective use of Prevena™ is paramount as in those cases that are not at high risk, Prevena™ may not be needed. However, in surgical cases with a high risk of incisional complications such as those mentioned before, the ability to control the incisional environment can be life-changing, for both the patient and the clinician. In this case, many factors lead to a positive outcome to include the patient's understanding and willingness to comply with all instructions as well as the clinician’s attention to detail both pre- and postoperatively as well as surgically. The decision to use Prevena™ for incisional management was confirmed as soon as the patient was recognized as a surgical challenge with a high risk for postoperative complications.

Abdominal procedures, particularly ventral hernia repairs with abdominal wall reconstruction, as well as those combined with excision of subcutaneous excess (panniculectomy) are fraught with high rates of wound healing complications. Panniculectomy involves the removal of the excess skin and soft tissue that extends beyond the pelvis. This procedure alone has a high incidence of complications including incisional dehiscence, infection, and seroma. Ventral hernia repair in a patient with morbid obesity also carries a high complication rate. In fact, those patients with a BMI >50.6 are considered superobese and have reoperation rates reported as high as 33% for wound-healing complications [11]. Furthermore, combining surgical procedures generally carries an increased risk of postoperative complications. The combination of panniculectomy with abdominal wall reconstruction may compromise blood flow to the remaining skin that may contribute to the additional high complication rate of these procedures.

The use of negative pressure over a closed incision, since described in 1997, has become increasingly popular given the current economic challenges in health care. Preventing a complication is always preferred over treating a complication once it has occurred. Additionally, the cost of these complications is noteworthy and cannot be ignored.

The patient presented fulfills the required criteria for high risk, specifically the indications for Prevena™ as described by the FDA.

Prevena™ has been described to offer several advantages to assist with the incisional environment. The reduction of lateral tension, edema control, and reduction of bacterial burden all likely played a role in the successful management of this patient. Clinical benefits have been reported to include reduction in infection, hematoma, and seroma [6-11]. Additionally, skin perfusion has been shown to improve with negative pressure incisional management in cardiac surgery [12]. This optimization of perfusion to the skin flaps may have played an even greater role in this case and should not be overlooked in patients undergoing panniculectomy where perfusion of the flaps is critical to a successful outcome.

I propose that ciNPT with Prevena™ should not be used in every surgical patient. However, every patient undergoing panniculectomy, and particularly those undergoing panniculectomy combined with abdominal wall reconstruction should be considered for ciNPT with Prevena™ and/or Prevena Plus™ 125 as a preferred dressing of choice for postoperative management. In this case, ciNPT assisted in a patient outcome that was successful and enabled a speedy recovery.

## Conclusions

ciNPT with Prevena™ and/or Prevena Plus™ is known to assist with edema control, reducing bacteria burden, and lateral tension that are beneficial to wound healing. Panniculectomy combined with abdominal wall reconstruction carries an even greater risk of postoperative complications than panniculectomy alone. Prevena™ incisional management has been shown to effectively reduce the risk of wound-healing complications in a variety of procedures. However, the ability to improve perfusion of the skin flaps that may be compromised given the extensive undermining and extent of excision inherent with these procedures may be an additional benefit of Prevena™.

I propose that while Prevena™ should not be used in every surgical patient and recommend consideration of ciNPT with Prevena™ and/or Prevena Plus™ 125 in every morbidly obese patient undergoing panniculectomy, and particularly morbidly obese patients undergoing panniculectomy combined with abdominal wall reconstruction. While more data on this is needed, ciNPT with Prevena™ and/or Prevena Plus™ 125 may become the preferred dressing of choice for postoperative incisional management in this challenging patient population.
